# Recombination Resulting in Virulence Shift in Avian Influenza Outbreak, Chile

**DOI:** 10.3201/eid1004.030396

**Published:** 2004-04

**Authors:** David L. Suarez, Dennis A. Senne, Jill Banks, Ian H. Brown, Steve C. Essen, Chang-Won Lee, Ruth J. Manvell, Christian Mathieu-Benson, Valentine Moreno, Janice C. Pedersen, Brundaban Panigrahy, Herman Rojas, Eric Spackman, Dennis J. Alexander

**Affiliations:** * Southeast Poultry Research Laboratory, Athens, Georgia, USA; † National Veterinary Services Laboratories, Ames, Iowa, USA; ‡ Veterinary Laboratories Agency–Weybridge, New Haw, Addlestone, Surrey, United Kingdom; § Ministerio de Agricultura, Santiago, Chile

**Keywords:** Influenza, poultry, orthomyxoviridae infections

## Abstract

Influenza A viruses occur worldwide in wild birds and are occasionally associated with outbreaks in commercial chickens and turkeys. However, avian influenza viruses have not been isolated from wild birds or poultry in South America. A recent outbreak in chickens of H7N3 low pathogenic avian influenza (LPAI) occurred in Chile. One month later, after a sudden increase in deaths, H7N3 highly pathogenic avian influenza (HPAI) virus was isolated. Sequence analysis of all eight genes of the LPAI virus and the HPAI viruses showed minor differences between the viruses except at the hemagglutinin (HA) cleavage site. The LPAI virus had a cleavage site similar to other low pathogenic H7 viruses, but the HPAI isolates had a 30 nucleotide insert. The insertion likely occurred by recombination between the HA and nucleoprotein genes of the LPAI virus, resulting in a virulence shift. Sequence comparison of all eight gene segments showed the Chilean viruses were also distinct from all other avian influenza viruses and represent a distinct South American clade.

Influenza viruses are segmented, negative-sense, single-stranded RNA viruses of the family *Orthomyxoviridae* and are divided into the genera *Influenzavirus A*, *B* and *C*. However, only type A influenza viruses have been known to cause natural infections of birds. Type A influenza viruses are further divided into subtypes based on antigenic relationships of the hemagglutinin (HA) and neuraminidase (NA) surface glycoproteins. To date, 15 unique HA subtypes (H1–H15) and nine unique NA subtypes (N1–N9) have been recognized. Each virus has one HA and one NA protein, potentially in any combination. Viruses of all HA and NA subtypes with most possible combinations of the HA and NA subtypes have been isolated from avian species.

Influenza A viruses infecting chickens and turkeys are usually at one of two extremes of virulence. Highly pathogenic avian influenza (HPAI) viruses cause a systemic disease with rapid death in chickens and turkeys, which often approaches 100%. Low pathogenic avian influenza (LPAI) viruses cause a localized infection with little or no disease unless exacerbated by other organisms or poor environmental conditions. To date, all HPAI isolates have been of the H5 or H7 subtypes, although not all H5 or H7 subtype viruses cause HPAI.

Although the virulence of AI viruses for birds is a polygenic trait, the virulence factor is correlated with the hemagglutinin cleavage site. For all influenza A viruses, the hemagglutinin glycoprotein is produced as a precursor, HA0, which requires posttranslational cleavage by host proteases before it is functional and virus particles are infectious (*1*). All HPAI viruses examined to date have had a motif with multiple basic amino acids (arginine and lysine) at the HA0 cleavage site. In contrast, the cleavage motifs of LPAI viruses typically have only two basic amino acids, at positions –1 and –4 from the cleavage site for the H5 and at positions –1 and –3 for the H7 subtype (*2*). This difference appears to have a direct influence on viral virulence as LPAI viruses are limited to cleavage by host proteases such as trypsin-like enzymes and are thus restricted to replication at sites in the host where such enzymes are found, i.e., the respiratory and intestinal tracts. Whereas the multiple basic amino acids at the HA0 cleavage sites of HPAI viruses, either as a result of insertion or substitution (*2*–*4*), allows the HA0 precursor to be cleavable by ubiquitous host proteases (*5*). As a result the HPAI viruses are able to replicate systemically, damaging vital organs and tissues, which results in severe disease and death (*1*).

Viruses of the H5 or H7 subtype isolated from free-living birds are almost invariably of low pathogenicity for poultry. With the exception of a large die-off of terns in South Africa in 1961 (*6*), from which A/tern/South Africa/61 (H5N3) was isolated, HPAI virus isolations from free-living birds have been associated with contact with infected poultry, usually as a result of surveillance of birds trapped or found dead on infected poultry farms. In addition, results of phylogenetic studies of H7 subtype viruses indicate that HPAI viruses do not constitute a separate phylogenetic lineage or lineages but appear to arise from low pathogenic strains (*7*–*9*). This finding is supported by the in vitro selection of mutants virulent for chickens from an avirulent H7 virus (*10*) and the emergence of HPAI virus from an LPAI virus isolated from swans after repeated passage in chickens (*11*).

These findings conform to the theories of the molecular basis for the mutation of avian influenza subtype H5 and H7 viruses from low to high virulence in poultry put forward by Garcia et al. (*12*) and Perdue et al. (*13*). Essentially they propose that spontaneous duplication of purine triplets results in the insertion of basic amino acids at the HA0 cleavage site and that this occurs due to a transcription fault by the polymerase complex. As pointed out by Perdue et al. (*13*) this mechanism is clearly not the only means by which HPAI viruses arise, as some appear to result from nucleotide substitution rather than insertion, while others have insertions without repeating nucleotides. Attempts to assess the minimum requirements to confer virulence for H7 avian influenza viruses have used site-directed mutagenesis of the cleavage site of cloned HA followed by in vitro expression to examine the effect that progressive amino acid changes at each position of the cleavage site would have upon cleavability (*3*,*14*,*15*). For some H7 influenza viruses, the tetrapeptide motif R-X-R/K-R at the cleavage site leading up to the cleavage point is sufficient for cleavage by furin-like proteases, and variants with cleavage sites that do not conform to this motif are not pathogenic for chickens (*3*). However, motifs that differ from this standard have been observed from natural outbreaks of H7 HPAI (*16*).

Between 1959 and the end of 2001, a total of 18 primary outbreaks (10 H7 and 8 H5) of HPAI in poultry were reported (*17*). However, the geographic distribution of these outbreaks was not uniform across the world (five were in the British Isles; five in Australia; three in areas in Europe other than British Isles; and one each in Pakistan, Hong Kong, Canada, United States, and Mexico). Until 2002, no influenza virus had been isolated in poultry or wild birds in the continent of South America. In May 2002, a LPAI virus of H7N3 subtype was isolated from a broiler breeder flock in Chile, and in June, an HPAI virus of the same subtype was obtained from the same flock (*18*). We describe the characterization of the LPAI and HPAI isolates obtained in Chile with particular reference to the HPAI virus, which, while having a 10–amino acid (aa) insert at the HA0 cleavage site, does not conform to the dogma that a -R-X-R/K-R*G-L-F- motif is a prerequisite for HPAI viruses. We also present evidence that the 10-aa insert present in the HPAI viruses is the result of recombination between the HA and the nucleoprotein genes.

## Materials and Methods

### Viruses

As a result of a disease outbreak in chickens in Chile (*18*), 13 hemagglutinating isolates (number 176822 obtained in May 2002 and numbers 4322, 4325, 4345, 4346, 4347, 4348, 4418, 4458, 4957, 4966, 4968, and 4977 obtained in June 2002) were submitted to Office of International Epizooties (OIE) reference laboratories for characterization. All viruses were propagated in the allantoic and amniotic cavities of 10- to 11-day-old embryonated specific pathogen free (SPF) chicken eggs for 48 to 72 h at 35°C to 37°C. Viruses submitted as isolates from Chile and other sources were passaged once in SPF eggs after receipt.

### Virus Characterization

Subtype identification was done by hemagglutination-inhibition (HI) and neuraminidase-inhibition (NI) tests by using polyclonal chicken antisera against a panel of influenza A reference strains, which had been prepared in SPF chickens. Virulence was assessed by the standard intravenous pathogenicity (IVPI) test by using 6-week-old SPF chickens and the standard pathotyping test by using 4- to 6-week-old SPF chickens (*19*,*20*). Both tests give a standard volume of virus intravenously to SPF chickens, but the IVPI test characterizes both illness and how many days the chickens remain alive to produce an index of virulence from 0 to 3. An IVPI index of >1.2 or death in chickens of >75% in the standard pathotyping test indicates a highly pathogenic avian influenza virus. Representative low and highly pathogenic viruses were also tested for their ability to replicate in chicken embryo fibroblast cells with or without trypsin (0.25 μg/mL) in the cell culture media.

### Molecular Cloning and Sequencing of Influenza Genes

RNA from the isolates examined in this study was extracted with either the Trizol LS reagent (Invitrogen, Carlsbad, CA), the RNeasy mini kit (Qiagen, Valencia, CA), or the QIAmp Viral RNA mini kit (Qiagen) from infectious allantoic fluid from embryonated chicken eggs before reverse transcriptase–polymerase chain reaction (RT-PCR) amplification. The RT-PCR amplification was performed with either a one-step or a two-step RT-PCR reaction. The one-step reaction used the Onestep RT-PCR kit (Qiagen) with incubation steps of 45°C for the PA, PB1, and PB2 genes and 50°C for the other genes for 30 min, and 95°C for 15 min and PCR incubation steps of 30 cycles of 53°C annealing for the PA, PB1, and PB2 genes and 56°C annealing for the HA, NA, M, NP genes for 15 s, 72°C extension for 60 s, and 94°C denaturation for 30 s. For the amplification of the nonstructural and matrix gene segments, primers in the 5′ and 3′ noncoding region of the RNA segment were used to amplify the complete coding sequence to be used for direct sequencing. The N3 gene segment was RT-PCR amplified in two parts and used for direct sequencing. Electrophoreses was performed on HA, NP, PB1, PB2, and PA PCR product, amplified with specific primers from the noncoding sequence, in a 1% agarose gel, and the products were extracted with the Qiaquick gel extraction kit (Qiagen) and cloned by using the pAMP ligation independent cloning system (Invitrogen). Colonies were screened by using PCR with internal primers; positive cultures were grown overnight, and plasmid was extracted using the Qiaprep spin miniprep kit (Qiagen). Alternatively, a two-step RT-PCR amplification with the Vgen primer (5′ AGCAAAAGCAGG) with MMLV reverse transcriptase (Promega, Madison, WI) and PCR with gene-specific primers was performed. For both direct PCR sequencing and plasmid sequencing, the ABI PRISM Bigdye terminator sequencing kit (Perkin Elmer, Foster City, CA) was used, and the reactions were run on ABI 3700 or 310 automated sequencers (Perkin Elmer).

### Sequence and Phylogenetic Analysis

The sequencing information was compiled with the Seqman II program (DNASTAR, Madison, WI), and nucleotide sequences were aligned with sequences from the influenza sequence database with the Megalign program (DNASTAR, Madison, WI) by using the Clustal V alignment algorithm. Pairwise sequence alignments were also performed in the Megalign program to determine sequence similarity between A/chicken/Chile/176822/02 and other published sequences for each gene segment. The origin of a 30-nt insertion at the HA cleavage site was determined by using the best-local-homology rapid search procedure (BLAST) against GenBank sequences. Phylogenetic comparisons of the aligned sequence for each gene segment were generated by using either the maximum parsimony method with 100 bootstrap replicates in a heuristic search with the PAUP 4.0b10 software (Sinauer Associates, Inc, Sunderland, MA) or with the maximum likelihood by using the PHYLIP phylogenetic inference package, version 3.57c (*21*) with transition/transversion ratios calculated by PUZZLE (*22*). Sequence data was submitted to GenBank with accession no. AY303630-AY303666.

## Results

### Virus Characterization

All 13 hemagglutinating isolates were identified as influenza A viruses of the H7N3 subtype. Six of the 7 isolates, 4322, 4325, 4418, 4957, 4968, and 4977, tested by the IVPI test were shown to be HPAI viruses with indices between 2.43 and 3.00. An IVPI of >1.2 is classified as a highly pathogenic avian influenza virus. Isolate 176822 was characterized as LPAI with an IVPI index of 0.00. Similar results were observed in the standard pathotyping test. The H7N3 virus was reisolated from chickens that died after intravenous injection with isolates 4322 and 4975. The amino acid sequence of the HA cleavage site was determined and shown to be the same as the injected virus. The low pathogenic and two highly pathogenic Chilean viruses, 176822, 4322, and 4957, were grown in chicken embryo fibroblast cell culture with and without the addition of trypsin to the media. The low pathogenic virus, 176822, did not plaque without the addition of trypsin. The HPAI viruses, which included a representative with and without the additional lysine at the cleavage site (see HA0 sequence below), plaqued with and without the addition of trypsin.

### HA0 Sequence

The deduced amino acid sequence at the hemagglutinin cleavage site for the H7N3 virus of low pathogenicity isolated in May 2002, 176822, was PEKPKTR/GLF. All the HPAI viruses had a 10-aa insert (basic amino acids are underlined) at the HA cleavage site, but the insert varied by a single nucleotide between some of the isolates (PEKPKTCSPLSRCRETR*GLF [isolates 4322, 4418 and 4977] and PEKPKTCSPLSRCRKTR*GLF [isolates 4325, 4957 and 4968] that resulted in an aa change from glutamic acid to lysine.

The insertion at the HA cleavage site is unlike any previously reported, and a BLAST search of this nucleotide sequence showed the most closely related sequence in the GenBank database was the nucleoprotein (NP) gene of A/gull/Maryland/704/77 at position 1268–1297, with 28 of 30 nucleotide identities. Nucleotide sequencing of the NP gene of the HPAI Chilean viruses 4077, 4346, 4957 and the LPAI virus 176822 indicated a 100% nucleotide sequence identity of the HA cleavage site insertion with the deduced amino acid sequence CSPLSRCRET. Three other conservative amino acid changes in the hemagglutinin were different between the LPAI and all the HPAI viral sequences including at position 38 A>T, position 141 D>N, and position 146 A>T. None of these changes involved potential N-linked glycosylation sites, and no additional potential N-linked glycosylation sites were observed in the Chilean viruses.

### Phylogenetic Analyses

**Figure 1:**
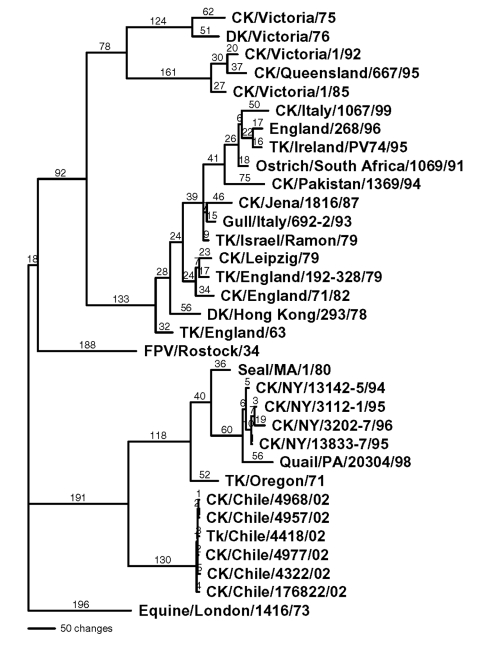
Phylogenetic tree of the hemagglutinin subtype 7 nucleotide sequence, which includes both low pathogenic and highly pathogenic avian influenza viruses from Chile. Representative avian and equine H7 influenza gene sequences are also included. The tree was generated with PAUP 4.0b4 computer program with bootstrap replication (500 bootstraps) and a heuristic search method. The tree is rooted to A/Equine/London/1416/73, and branch lengths are included on the tree. Standard two-letter postal codes are used for states in the United States. TK, turkey; CK, chicken; DK, duck; and FPV, fowl plague virus.

The complete coding sequence for all eight gene segments from the low pathogenic isolate (A/chicken/Chile/176822/02) and one of the highly pathogenic isolates (A/chicken/Chile/4957/02) was determined. Additional genes from other isolates were also sequenced and used for comparison. The low pathogenic isolate, A/chicken/Chile/176822/02, was chosen as the reference isolate for comparison with the influenza database, and pairwise sequence analyses were performed to identify the most closely related isolates for each gene segment. The closest nucleotide sequence similarities ranged from 82.1% for the HA gene to 96.3% for the matrix gene. The amino acid sequence similarity was much higher for all gene segments (Table). Phylogenetic trees were constructed from nucleotide sequences for all eight gene segments, and in general the Chilean isolates clustered most closely with North American avian isolates. Although the most closely related gene segments to the PB2 and PB1 gene segments were viruses isolated from swine, in both cases the swine isolates were thought to be the result of a recently introduced or reassorted avian influenza virus in North America (*23*,*24*). Exceptions were the nucleoprotein (Figure 1) and polymerase acid protein (PA) genes, which were most closely related to H7N7 equine influenza viruses which had internal genes derived from the equine type 2 H3N8 viral lineage.

**Table:** Comparison of individual gene segments of A/chicken/Chile/176822/02 with influenza viral genes from GenBank with the highest sequence similarity

Gene	Nucleotide similarity	%	Amino acid similarity	%
PB2	Swine/NC/98225/01	82.2	Shorebird/DE/9/96	97.9
PB1	Swine/Ontario/01911-1/99	93.6	Gull/MD/704/77	98.7
PA	Equine/London/1416/73	87.4	TK/MN/833/80	98.3
H7	Chicken/NY/13142-5/94	82.1	Seal/MA/1/80	90.7
NP	Equine/London/1416/73	90.0	Swine/Ontario/01911-1/99	98.4
N3	Turkey/Oregon/71	86.5	Turkey/Oregon/71	93.4
Matrix, M1	Turkey/Oregon/71	96.3	Many Isolates	100.0
M2			Many Isolates	99.0
NS, NS1	Turkey/Canada/63	91.2	Turkey/England/50-92/91	98.3
NS2			Chicken/Mexico/31381-1/94	95.8

**Figure 2:**
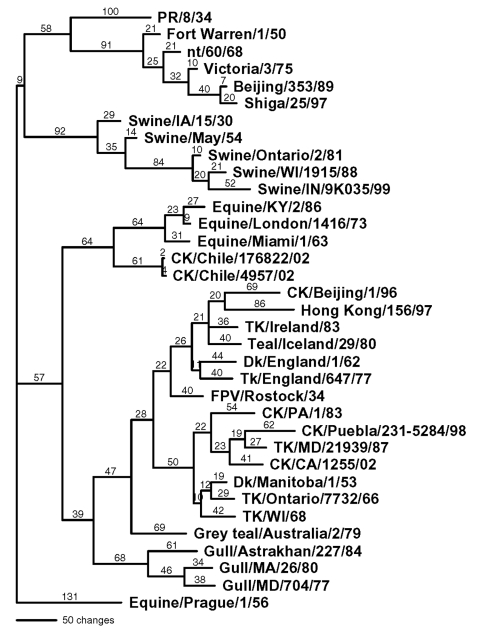
Phylogenetic tree of the nucleoprotein nucleotide sequence, which includes both low pathogenic and highly pathogenic avian influenza viruses from Chile. Representative avian, human, swine, and equine influenza gene sequences are also included. The tree was generated with PAUP 4.0b4 computer program with bootstrap replication (500 bootstraps) and a heuristic search method. The tree is rooted to A/Equine/Prague/1/56, and branch lengths are included on the tree. Standard two-letter postal codes are used for states in the United States. TK, turkey; CK, chicken; DK, duck; and FPV, fowl plague virus. For isolates without a species, it is assumed to be an isolate from a human.

For all gene segments, the Chilean viruses formed a distinct subgroup from other influenza viruses. Analyses of the HA gene indicated that all the Chilean H7N3 viruses were closely related and that the HPAI viruses emerged from the LPAI virus. The H7 phylogenetic tree, when using Equine/Prague/1/56 as the outgroup, shows the avian isolates divided into two main branches that are further subdivided into geographically defined groups (Figure 2). One branch includes the North American avian and the Chilean viruses, and the second branch includes the Eurasian avian and Australian avian viruses. Although the Chilean viruses are most like avian viruses of North American origin they are distinctly different from these viruses and form a unique branch on the tree.

**Figure 3:**
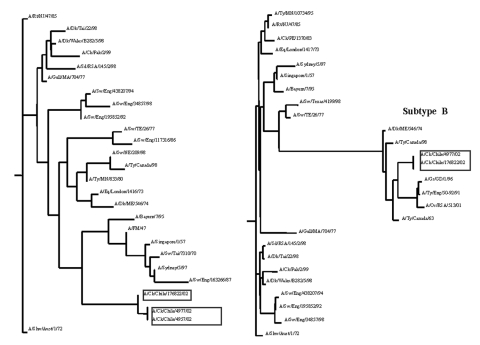
Unrooted phylograms of partial nucleotide sequences of the PB2 and NS genes of selected influenza A viruses including those from poultry in Chile in 2002 (indicated in boxes). Nucleotides 14–188 of PB2and 50–481 of NS were used for the analyses. The lengths of the horizontal lines are proportional to the number of nucleotide differences.

For the N3 tree little sequence data were available to make meaningful observations about the phylogeny. In conserved internal proteins like the matrix and nucleoprotein genes, differences in North American avian and Eurasian avian influenza viruses can be observed at the nucleotide level, but almost all tree topology structure is lost when comparing the same isolates in phylogenetic trees based on amino acid sequence. This observation is common for avian influenza viruses which often have high sequence conservation at the amino acid level, but with large differences at the nucleotide level (*25*). The results of nucleotide analyses are presented for PB2 and NS genes as unrooted phylograms (Figure 3). Of the genes examined for the six different isolates, little sequence difference was observed between the low pathogenic and highly pathogenic viruses except for the H7 gene segment where an insertion of 30 nucleotides was present at the HA cleavage site.

## Discussion

Sequence analysis shows that the Chilean isolates are unique. None of the eight genes are closely related at the nucleotide level to any other genes in the available sequence databases. For example, the nucleotide sequence of the hemagglutinin gene was 17% divergent from the most closely related virus in GenBank. At the nucleotide level they were more closely related to North American avian or avianlike viruses for six of the eight influenza gene segments. Even the NP and PA genes, which clustered most closely with equine viruses, were more closely related to the North American avian lineage of viruses than any other avian lineage. However, all eight genes were also uniquely distinct with relatively long branch lengths from the most closely related virus. For the H7 hemagglutinin gene, one of the most variable influenza genes, at both the nucleotide and amino acid level, five distinct lineages can be identified, including equine type 1, Eurasian, Australian, North American, and Chilean. The Eurasian and Australian avian viruses are more closely related to each other than the other lineages, but they still differ by 15%–20% at the nucleotide level. A similar relationship exists between the North American and the Chilean lineages, with nucleotide sequence differences of about 20%. For avian influenza viruses, geographic influences rather than the host species infected are usually more important in determining the phylogenetic lineage to which the virus belongs. However, exceptions to this rule of geographic origin have been observed infrequently with avian influenza viruses from wild birds (*26*).

What is not clear is how prevalent avian influenza viruses are in the wild bird population in South America since avian influenza has not been isolated. This may reflect the low degree of sampling or the low prevalence of infection in wild birds. However antibodies to H1N1 and H3N2 have been reported in wild and domestic birds in Brazil (*27*).

Phylogenetic analyses of the viruses isolated during the H7N3 outbreak of avian influenza in Chile in 2002 indicate that the HPAI viruses emerged from the LPAI virus or a close common ancestor. The HA cleavage site motifs of the Chile HPAI viruses do not conform to the recognition motif -R-X-R/K-R*G-L-F- for the furin-like proteases that are reportedly responsible for allowing HPAI viruses to initiate systemic infections (*3*). In addition, the insertion of 10 aa could not have occurred by the viral RNA polymerase slippage mechanism proposed by Garcia et al. (*12*). It seems most likely that this insertion occurred by a RNA recombination event between the HA and NP gene of this virus. The evidence for this is the 100% nucleotide sequence homology between the 30 bases coding for the HA insert and nucleotides 1268–1297 of the NP for the LPAI virus and of the HPAI viruses sequenced. No palindromic sequences were observed on either side of the insert region, so the mechanism by which recombination occurred is not clear. Viruses with the CSPLSRCRKT amino acid insert are most likely to have evolved from those with CSPLSRCRET motif after recombination, since their NP genes only have the latter sequence.

The increase in virulence between viruses with and without the insert in this outbreak are readily apparent. The virus without the insert caused no illness or death in experimentally infected birds and had an IVPI of 0.0. Viruses with the insert caused severe disease and death in experimentally infected birds and had an IVPI in the range of 2.43–3.0. No correlation with an increased IVPI index was seen with isolates with the additional lysine at the HA cleavage site (range 2.53–3.0). Also, both viruses with the HA cleavage site insert were able to plaque in cell culture without the addition of trypsin to the media, but the virus without the insert could not. All highly pathogenic avian influenza viruses are believed to arise from low pathogenic precursor viruses. The mechanism of this conversion can be extremely variable, but has included both nucleotide substitutions or insertions at the hemagglutinin cleavage site. The Chilean HPAI isolates were unusual not only because of the size of the insert but also because the viruses were highly pathogenic with only three basic amino acids near the cleavage site (–1, –4, and –6 positions) for the first HPAI viruses in the outbreak, although a substitution occurred later in the outbreak, resulting in an additional basic amino acid at the –3 position. Other influenza viruses have been observed with 10 additional amino acids at the HA cleavage site including the equine type 1 (H7N7) viruses, with A/Equine/Prague/56 as the prototype virus. These viruses have four basic amino acids at the cleavage site and can grow in cell culture without trypsin (*28*), a characteristic for avian viruses of the highly pathogenic phenotype. However, the H7N7 viruses are not considered to cause a systemic disease in horses, but they have been described as causing systemic infection in mice without prior adaptation (*29*). Also, when the H7 gene from A/Equine/London/1416/73 was reassorted with an avian influenza virus, the reassortant virus had a lethal phenotype in chickens (*30*).

The two in vitro examples of recombination in avian influenza viruses also involved nucleotide insertions at the hemagglutinin cleavage site. Both cases involved H7 influenza viruses, A/Turkey/Oregon/71 (TK/OR/71) (H7N3) and A/Seal/Massachusetts/1/80 (H7N7). An insert of 54 nucleotides, from 28S host ribosomal RNA, was inserted in A/Turkey/Oregon/71, and 60 nucleotides, from the nucleoprotein gene of the virus, was inserted into A/Seal/ Massachusetts/1/80. With in vitro experiments, virus variants of both viruses were selected that could plaque in cell culture without the addition of trypsin, and both showed an increased virulence in chickens. Experimental inoculations resulted in clinical signs suggestive of a systemic disease for A/Seal/Massachusetts/1/80 and a highly pathogenic phenotype for A/Turkey/OR/71 (*31*,*32*).

In conclusion, the influenza infections of poultry in Chile in 2002 were both the first reported isolations of influenza viruses in poultry in South America as well as the first HPAI outbreak. The viruses isolated showed several unique properties: 1) They formed a genetic group distinct from other influenza viruses but closest to North American viruses; 2) The HPAI viruses had a unique 10-aa insert at the cleavage site of the HA0 precursor protein; 3) Neither of the two forms of this insert conformed to the assumed minimum motif for high pathogenicity at the cleavage site of -R-X-R/K-R*G-L-F-; 4) The nucleotide sequence coding for the insert showed 100% homology with a region of the nucleoprotein gene indicating the insertion had occurred as the result of a recombination event.
